# Optimization of a centrifugal blood pump designed using an industrial method through experimental and numerical study

**DOI:** 10.1038/s41598-024-57019-9

**Published:** 2024-03-28

**Authors:** Kohyar Yazdanpanah-Ardakani, Hanieh Niroomand-Oscuii, Reza Sahebi-Kuzeh Kanan, Nasim Shokri

**Affiliations:** Department of Biomedical Engineering, Sahand University of Technology, Tabriz, Iran

**Keywords:** Ventricular assist device (VAD), Point-by-point method, Centrifugal pump, Blade profile, Engineering, Biomedical engineering, Cardiac device therapy

## Abstract

With improved treatment of coronary artery disease, more patients are surviving until heart failure occurs. This leads to an increase in patients needing devices for struggling with heart failure. Ventricular assist devices are known as the mainstay of these devices. This study aimed to design a centrifugal pump as a ventricular assist device. In order to design the pump, firstly, the geometrical parameters of the pump, including the gap distance, blade height, and position of the outlet relative to the blade, were investigated. Finally, the selected configuration, which had all the appropriate characteristics, both hydraulically and physiologically, was used for the rest of the study. The study of the blade, as the main component in energy transfer to the blood, in a centrifugal pump, has been considered in the present study. In this regard, the point-to-point design method, which is used in industrial applications, was implemented. The designer chooses the relationship between the blade angles at each radius in the point-to-point method. The present study selected logarithmic and second-order relations for designing the blade’s profile. In total, 58 blades were examined in this study, which differed regarding blade inlet and outlet angles and the relationship between angle and radial position. ANSYS CFX 17.0 software was utilized to simulate blades’ performances, and a benchmark pump provided by the US Food and Drug Administration (FDA) was used to validate the numerical simulations. Then, the selected impeller from the numerical investigation was manufactured, and its performance was compared experimentally with the FDA benchmark pump. A hydraulic test rig was also developed for experimental studies. The results showed that among the blades designed in this study, the blade with an input angle of 45° and an output angle of 55°, which is designed to implement a logarithmic relationship, has the best performance. The selected impeller configuration can increase the total head (at least by 20%) at different flow rates compared to the FDA pump.

## Introduction

Heart failure is recognized as one of the most mortal diseases in industrial countries^1^. Mechanical circulatory support devices have emerged as a primary treatment for patients with end-stage heart failure when medical treatments fail^1^. A wide variety of these devices have been proposed to provide circulatory support and recover natural heart function. In patients with partially impaired heart, Ventricular Assist Devices (VAD) are implemented to deliver adequate blood perfusion in patients without notably damaging the blood^1–7^. VADs are designed for Left or right and even both hearts; with this in mind, VADs are categorized as left ventricular assist device (LVAD), right ventricular assist device (RVAD) and Biventricular assist device (BVAD). VADs are prescribed with four therapeutic aims; bridge to bridge (in which a time span is provided to choose the appropriate therapy), bridge to recovery (In which VAD is prescribed with the perspective of recovering normal heart functionalities without transplanting donor heart), bridge to transplant (in which defected heart functionalities is improved by VAD, until a donor heart is found) and bridge to therapy (in which VAD is prescribed as final therapy)^2^.

Through the evolvement of VAD, different generations of VAD had been proposed which can be categorized as three generations. First generation are volume-displacement VADs. They consist of a chamber which is passively filled by blood and compressed electrically or pneumatically^8–11^. By applying periodic change in chamber space, a pulsatile flow is generated. Despite that Volume-displacement VADs behave more similar to natural heart, they have disadvantages such as large sizes (which make them not fully implantable), noisy operating mode (noises due to mechanical heart valve operation) and limited durability^12–16^.

Second- and third-generations are rotary pumps. Against first generation pump, rotary pumps produce continuous flow. The kinetic energy needed for increasing blood flow and pressure, transfers by a rotating part within the pump called Impeller. In the structure of an impeller, there are some blades with specific geometry by which they have a prominent impact on pump hydraulic performance. Bearing system in second generation is mechanical while it is contactless in third generation. It is reported that rotary pumps have higher mechanical durability and smaller size than volume-displacement pumps^3–17^.

For deciding to insert a VAD, some parameters must be taken into account including patient’s quality of life after VAD insertion, the reliability and cost-effectiveness^9^. There are some other challenges which are in spot light of engineers such as minimizing VADs size, prolonging its life and blood damage. Blood damage is another big challenge in designing blood-contact devices. The primary cause of blood damage is high shear which has been shown to cause hemolysis^9,10, 18, 19^. Release of hemoglobin into blood plasma due to complete rupture of red blood cell (RBC) membrane or formation of pores in RBC membrane is known as hemolysis^20^.

Computational fluid dynamics (CFD) is an investigational tool that can estimate flow fields variables such as velocities, pressures, and shear stresses using numerical techniques^21^. It is becoming increasingly important tool for the design and development of medical devices specifically VADs. It is primarily used to predict the hydraulic performance and flow distributions. Moreover it makes the modifications and optimization process of the device fast and with lower cost^22–27^. Over the last decade, CFD was implemented to predict hemolysis numerically in many researches. Generally, there are two types of numerical approaches available: Eulerian and Lagrangian approaches. In the Eulerian approach, the damage index is integrated over the entire computational flow domain whereas in the Lagrangian formulation the integration is along the flow path lines^20^.

While CFD modeling is increasingly used in designing medical devices involving blood flow, it lacks credibility due to inadequate validation. This has motivated the US Food and Drug Administration to initiates a benchmark blood pump to provide a comparison of numerical results with experimental data. The FDA pump was tested in multiple laboratories to provide experimental data and it is used as a reference of accuracy of the CFD application^28^. Many CFD studies on different LVADs are validated by comparing the results obtained from the simulation of the FDA benchmark pump using their proposed numerical simulation approach with the reported FDA experimental data^18,19, 29–36^.

A considerable amount of investigations have been conducted to understand the effects of components’ different geometries on LVAD’s flow characteristics. Consequently, Impellers, as the main effective part of LVAD for increasing fluid velocity, have been the focus of much attention. For instance, the impellers with airfoil geometry as blade profile, blade with splitters and different angles have been investigated in many pervious researches^37–50^. As new designs are developed, in an effort to improve the LVAD performance, there is a continuing need for computational analysis of their functionality^27^.

Manufacturing is another challenge in designing VADs. Cost, simplicity, and safety are the criteria of a preferred engineering design concept. Moreover, the concept must have a large degree of design freedom and limited manufacturing constraints^51^. The impeller’s geometry of the current VADs have many complexities which leads to their higher manufacturing cost. Paul et al.^52^ conducted a numerical research to find optimal geometries for shrouded impellers that can be machined with conventional machining processes from a single piece. However, their method had some limitations on the impeller’s blade angle^53^.

In this study we designed an impeller using specific industrial method (point-by-point method) which can be produced simply and fulfill the requirement of an LVAD. Considering the fact that industrial designing methods of impeller blades’ profile are not clearly presented in literature^54^, we initiate this research to investigate the feasibility of these method in designing an LVAD. To date the point-by-point method has not been applied to design LVAD impeller. We took the FDA benchmark blood pump as the reference and conduct the study by investigating the performance of our designed impellers in the housing of the FDA pump. These impellers are different with respect to their inlet/outlet angle. The impellers’ performance were evaluated numerically by a validated model. The CFD modeling was performed by ANSYS CFX17 package. Blood damage was numerically evaluated using MATLAB and CFD generated data. A test rig was constructed to assess the hydraulic performance of the impeller which had the highest performance among the proposed impellers.

## Materials and methods

The processes of designing blades, simulation and experimental test are presented in the following subsections.

### Impeller blades profile

**Figure 1:**
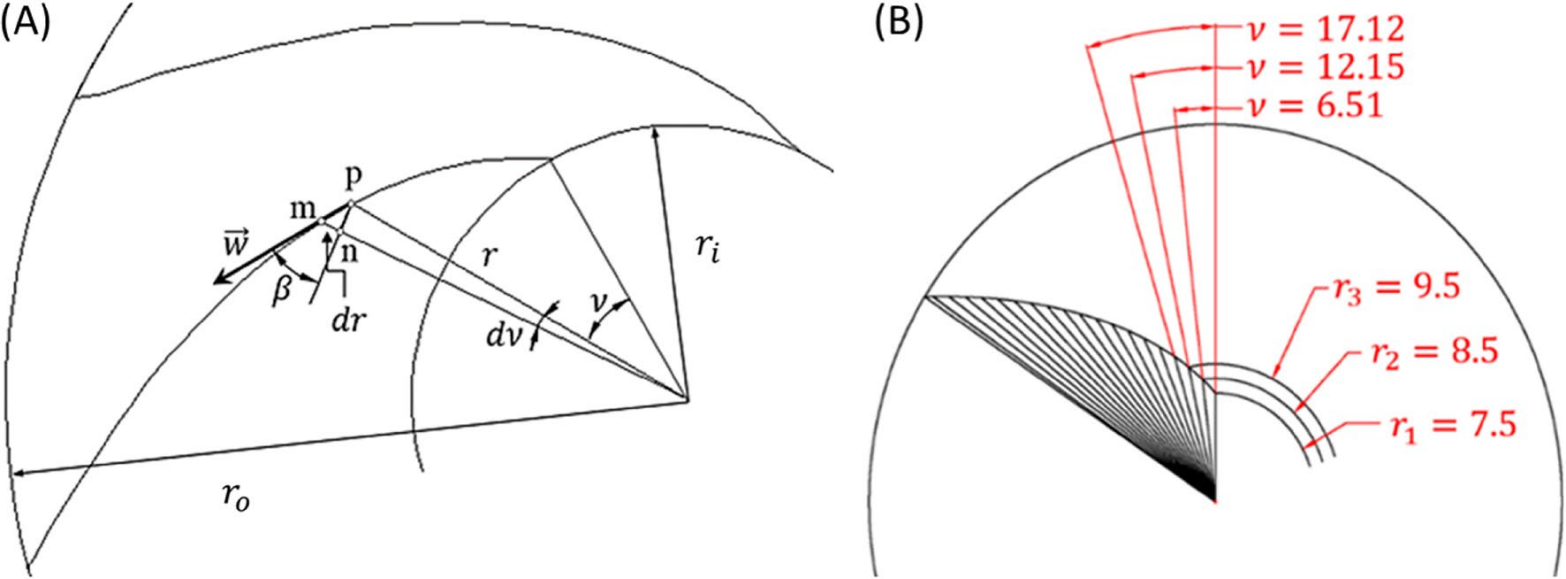
(**A**) Blade construction scheme. P, m and n are imaginary points, (**B**) constructing the blade profile using the data of Table 1.

For designing an efficient impeller, the impeller’s blade profile is of significant importance from the inlet to the outlet of the impeller. Modifying the impeller blades geometry leads to the better performance of centrifugal pumps^55^. If the blade angle changes smoothly with respect to the radial distance, a smooth flow will be created while it passes through the impeller. There are three methods for determining the blade profile. They are Simple-arc, double-arc and point-by-point methods. Simple-arc and double-arc concentrate on the conditions just at the point of inlet and outlet while the blades which are constructed with point-by-point method can take into account the requirement within the flow channel^56,57^. According to Fig. 1A, angle increments between two adjacent points is calculated as Eq. (1).

$$\left\{ {\begin{array}{*{20}c} {\overline{pn} = rd\nu } \\ {\overline{pn} = \frac{{\overline{mn} }}{\tan \beta }} \\ \end{array} \mathop \Rightarrow \limits^{{\overline{mn} = dr}} } \right. d\nu = \frac{dr}{{r\tan \beta }} \to ^{ } \nu = \frac{180}{\pi }\mathop \smallint \limits_{{r_{i} }}^{{r_{o} }} \frac{dr}{{r\tan \beta }},$$

1 $$\nu = \frac{180}{\pi }\mathop \sum \limits_{{r_{i} }}^{{r_{o} }} \frac{\Delta r}{{r\tan \beta }}.$$

$\beta$ is blade angle, $\overrightarrow{w}$ is the velocity vector, *r* is radial distance, *r*_*i*_ is blade’s inlet radial position, *r*_*o*_ is the blade’s outlet radial position, $\overline{pn }$ is the distance between point p and point n, $\overline{mn }$ is the distance between point m and point n, $\nu$ is wrap angle, $d\nu$ and $dr$ are change in wrap angle and radial distance, respectively. $\Delta r$ is the radial increment. According to Eq. (1), blade angle at different radius must be determined for defining the blade profile. Relationship between blade angles at different radiuses must be chosen by the designer^2^. In the current investigation both logarithmic and second order functions are used which are monotonic from *r*_*i*_ to *r*_*o*_ as Eqs. (2) and (3).

2 $$\beta =aLn\left(r\right)+b,$$

3 $$\beta =a{x}^{2}+b.$$

**Figure 2:**
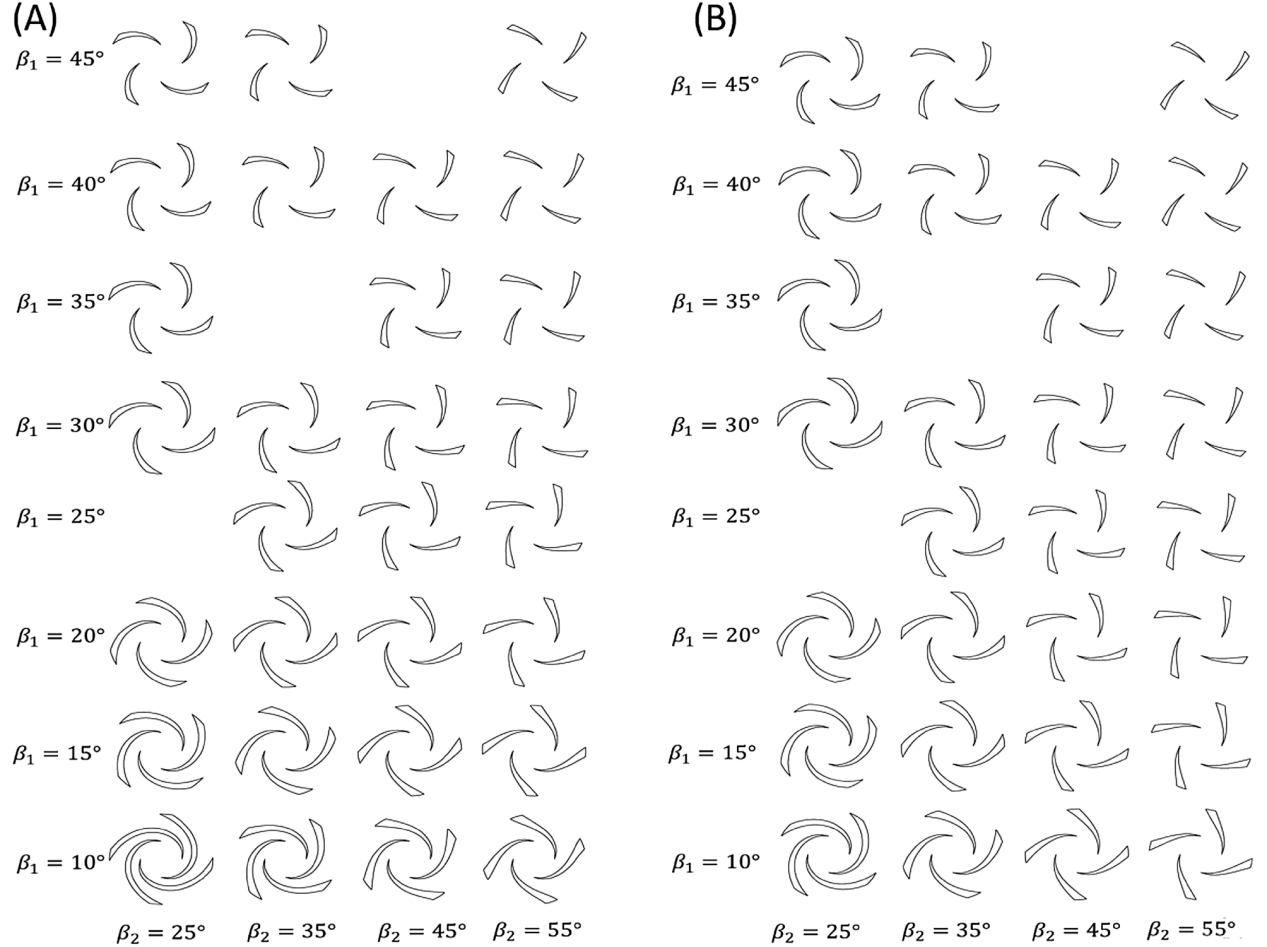
All the blades’ profile that are designed using point-by-point method with different inlet blade angle ${\upbeta }_{1}$ and outlet blade angle ${\upbeta }_{2}$, (**A**) second order function and (**B**) logarithmic function.

In which *a* and *b* are constants that calculated according to the inlet and the outlet angles. Table 1 represent the changes of blade angles for the blade with inlet blade angle ${\beta }_{1}=45$ and outlet blade angle ${\beta }_{2}=55$ and wraping angle with respect to radial distance calculated from Eq. (2). $\Delta r$ is chosen to be equal to 1. The blade profile using the data of Table 1 is shown in Fig. 1B. All the impellers that are designed using this method is shown in Fig. 2.

**Table 1:** Wrap angle for different radius considering Eq. (2), ${\upbeta }_{1}=45$ and ${\upbeta }_{2}=55$.

r (mm)	$\upbeta$(°)	$\upnu$(°)
7.5	45	
8.5	46.01	6.51
9.5	46.90	12.15
10.5	47.71	17.12
11.5	48.44	21.53
12.5	49.11	25.50
13.5	49.73	29.10
14.5	50.30	32.38
15.5	50.84	35.39
16.5	51.34	38.17
17.5	51.82	40.74
18.5	52.26	43.14
19.5	52.69	45.38
20.5	53.09	47.48
21.5	53.47	49.45
22.5	53.84	51.31
23.5	54.19	53.07
24.5	54.52	54.74
25.5	54.84	56.32
26	55	57.09

### LVAD model

**Figure 3:**
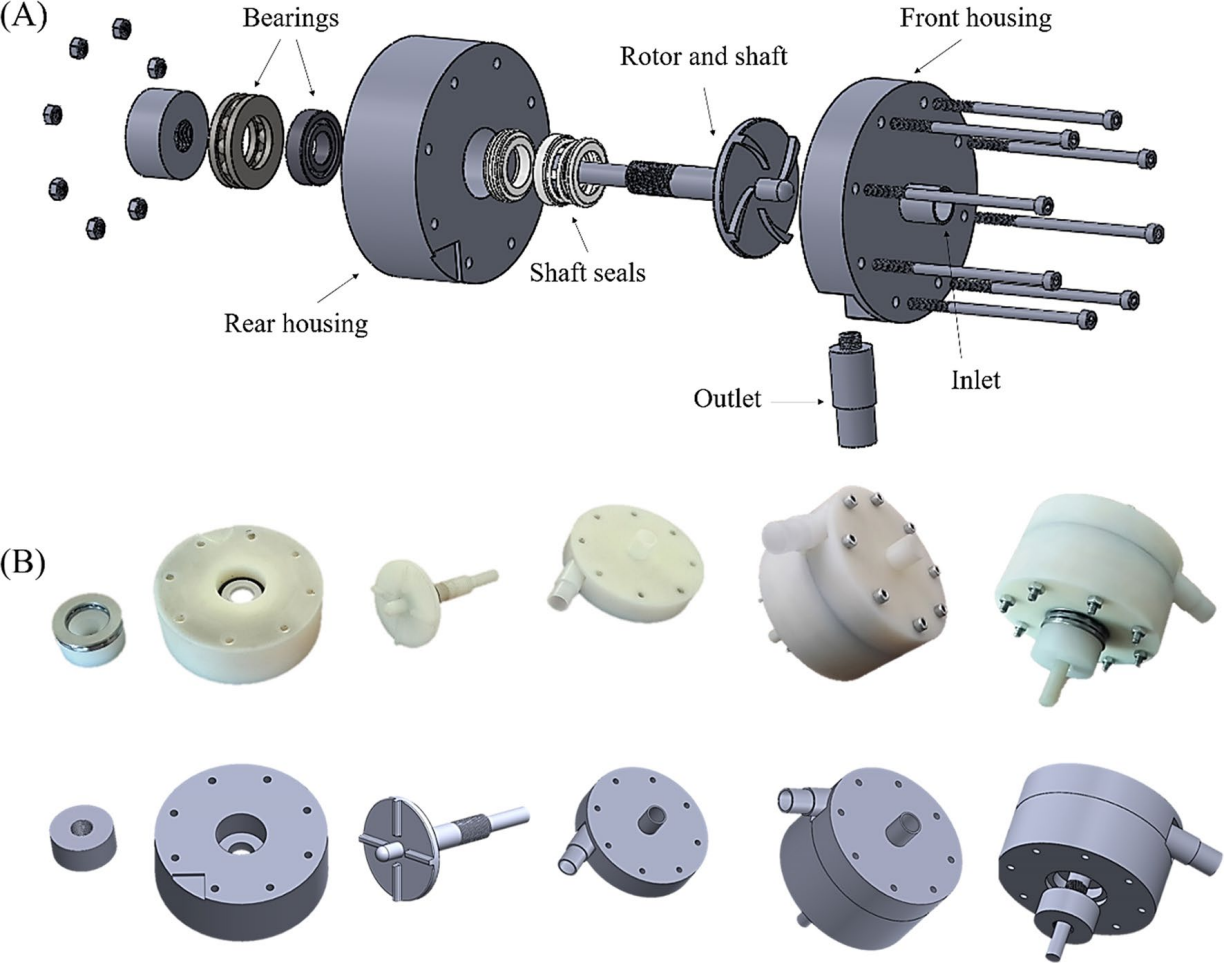
(**A**) 3D exploded view of the designed pump in this study, (**B**) main parts of the pump and the final assembly of the pump.

Figure 3 details a three-dimensional drawing of the LVAD. It consists of four parts: a front housing, a rear housing, an impeller and outlet port. All the parts were made from Polyamide (Fig. 3B). This material has enough strength to be used under load condition of a LVAD prototype. Moreover, it can be machined easily. The diameter of the impellers are 5.2 cm and the blades height are 3 mm. All of the parts have the same dimensions as the FDA pump except the impeller’s blades profile. For sealing the rotating parts a spring-loaded, polymer-filled PTFE seal was used. The seal is indented within the rear housing.

### Hydraulic test setup

**Figure 4:**
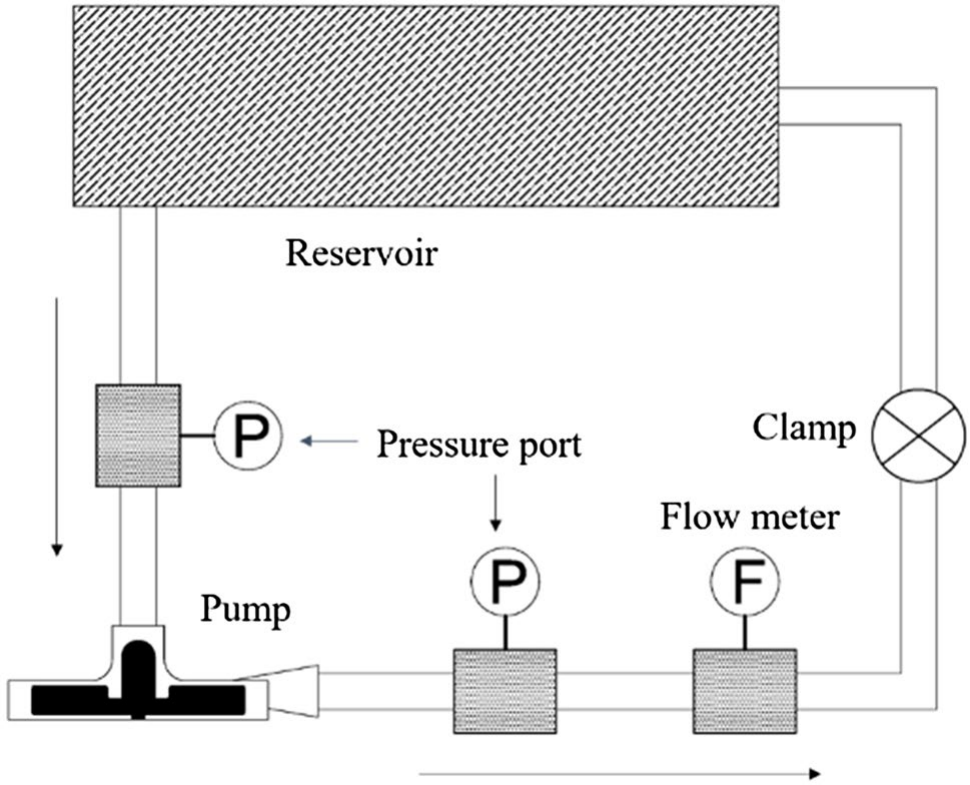
Hydraulic test setup.

The constructed hydraulic test setup contains a pump, measuring sensors (including two pressure transducers and a flow meter), controller box, a total of 2.5 m flexible PVC tubing (1/2 in ID), a clamp, an AC servo motor (ECMA-C20807RS, DELTA, Taiwan) and a reservoir. As shown in Fig. 4. The pressure sensors (PR-23RY/80710.34; KELLER AG, Winterthur, Switzerland) were mounted on the inlet and the outlet tubing of the pump. An electromagnetic flow meter (embedded in MEDTRONIC 550 Bio-console) was placed after the outlet pressure sensor. The clamp was used to adjust the flow rate. The reservoir was place at the height in order to facilitate the test loop debubbling. The results of the test are reported for blood with 1035 (kg/m^3^) density and 0.0035 (Pa s) viscosity.

### Governing equation

Governing equations without body forces of the Newtonian fluid flow for an inertial reference frame is given in Eqs. (3) and (4):

3 $$\nabla \cdot \overrightarrow{V}=0,$$

4 $$\rho \frac{D\overrightarrow{V}}{Dt}=-\nabla P+\mu {\nabla }^{2}\overrightarrow{V},$$

where $\overrightarrow{V}$ is velocity vector, $P$ is static pressure, $\mu$ is Viscosity and $\rho$ is density. In this work, blood was considered to be Newtonian fluid and its viscosity was $0.0035 (\text{Pa s})$; density was also 1035 kg/m^3^.

#### Turbulence modeling

According to the dimensions and the rotational speed of the impeller in LVADs, the flow could be considered as the turbulence flow pursuant to the Reynolds number definition^58^. The choice of the turbulence model depends on such considerations as the physics encompassed in the flow, the established practice for a specific class of problem, the level of accuracy required, the available computational resources, and the amount of time available for the simulation. It is an unfortunate fact that no single turbulence model is universally accepted as being superior for all classes of problems^59^. Currently, one of the most prominent two-equation models is $k-\omega$ based models of Menter. The based Shear-Stress-Transport (SST) model was designed to give a highly accurate prediction of the onset and the amount of flow separation under adverse pressure gradients by the inclusion of transport effects into the formulation of the eddy-viscosity^60^. This can result in a major improvement in terms of flow separation predictions. The superior performance of this model has been demonstrated in a large number of validation studies^61^. So $k-\omega SST$ model was applied for modeling turbulence.

#### Hemolysis

Red blood cells experience varying shear stresses while passing through the LVAD. Lagranian tracking method is applied for assessing the accumulative shear stress. To estimate the hemolysis, a power-law model presented by Heuser et.al could be considered^20^. They represent the relation between the hemolysis index, the shear stress and the exposure time, as shown in Eq. (3). This relationship includes turbulent and viscous stresses.

5 $$\frac{dHb}{Hb}=1.8\times {10}^{-6}\cdot {\tau }^{1.991}\cdot {T}^{0.765},$$

where $dHb$ is the amount of free hemoglobin of the blood, $\tau$ is scalar shear stress which is calculated using Eq. (4) and $T$ is exposure time. By applying the integral approach on Eq. (3) over a streamline, the blood damage index (D) for a single streamline is expressed as represented in Eq. (5).

6 $$\tau ={\left[\frac{1}{6}\sum {\left({\tau }_{ii}-{\tau }_{jj}\right)}^{2}+\sum {\tau }_{ij}^{2}\right]}^\frac{1}{2},$$

7 $$D=\int \limits_{inlet}^{outlet}1.8\times {10}^{-6}\cdot {\tau }^{1.991}\cdot {dT}^{0.765}.$$

Finally, by taking average over sufficient number of streamlines, hemolysis index (HI) for the blood passing through the LVAD is calculated using Eq. (5).

8 $$HI=\sum_{inlet}^{outlet}1.8\times {10}^{-6}\cdot {\tau }^{1.991}\cdot {dT}^{0.765}.$$

The present method of estimating blood damage was implemented by Song et.al and the value of HI estimated by their method is of the same order of magnitude as indices approximated for clinical VADs (which is reported to be 0.04 to 0.06), giving credence to their approach^1–3^. Meanwhile, a widely accepted blood damage modeling equation which can accurately predict the absolute level of hemolysis, has not been proposed yet^4,5^. Although the absolute values of HI calculated by CFD predictions are not credible, it can be used for comparing between different model’s blood damage, relatively. As blood damage caused by LVAD is a critical parameter, the present study compare HI between different models along with fulfillment of hydraulic parameters required.

### Numerical solution

ANSYS CFX is widely used for simulating the performance of VADs which approve that if the VAD model is well prepared according to numerical concerns, it can deservedly characterize the performance of small size blood pumps^19,29–34, 62–66^. In the proceeding subsections, the preparation of numerical model will be explained.

#### Grid generation

**Figure 5:**
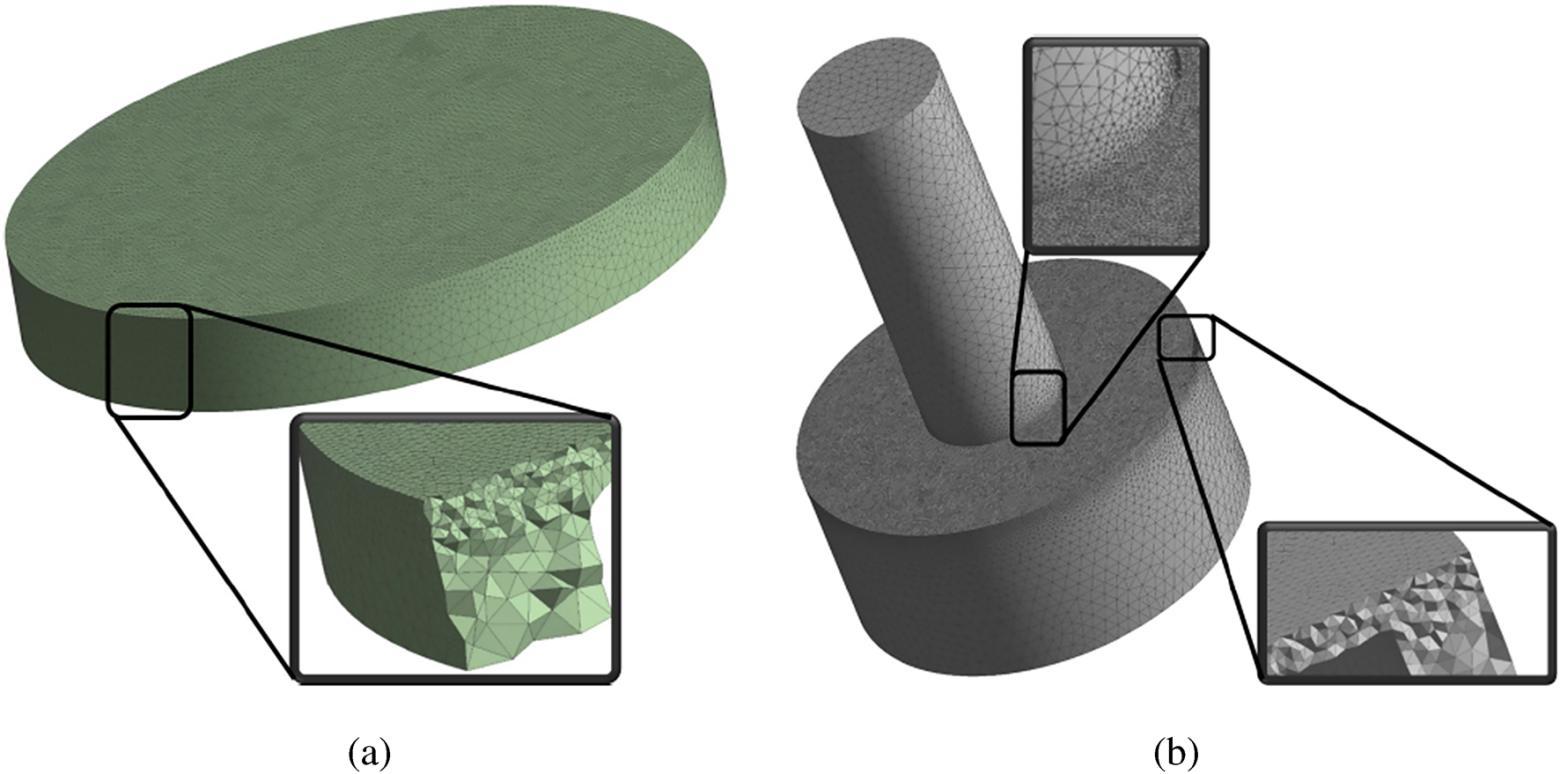
Mesh density illustration, (**a**) rotary and (**b**) stationary.

**Figure 6:**
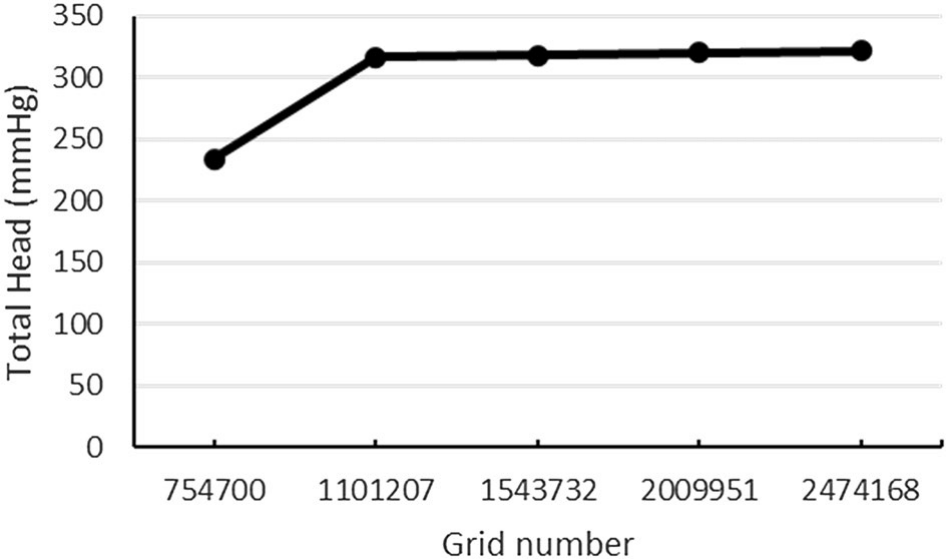
The error percentage for the total head of chosen mesh configuration.

Due to the existence of the high shear stress in a small area between the impeller and the pump casing, it is necessary to create dense mesh in the area; both casing and rotating domains are depicted in Fig. 5. The total number of elements was 1.5 million elements of the linear tetrahedral, which has been selected through performing mesh independency calculations for FDA pump at the condition number 5 (6 (l/min) and 3500 (rpm)). The error percentage for the total head of chosen mesh configuration is 0.7% compared to the finer mesh, as shown in Fig. 6.

#### Boundary conditions

In the present study, pressure and volume flow rate boundary conditions were used for the inlet and outlet, respectively. The volume flow rate in the outlet was chosen equal to 5 l/min^67–70^ The total head of centrifugal pumps are almost constant^35^; accordingly they are not so much sensitive to inlet pressure, though the Pressure in the inlet was chosen to be 40 mmHg which is left Intraventricular average pressure. For many problems, it may be possible to refer the entire computational domain to a single moving reference frame. For more complex geometries, such as present geometries, it may not be possible to use a single reference frame (SRF); therefor, the problem must be broken up into multiple zones (stationary zone and rotational zone), with well-defined interfaces between the zones^59^ Outer walls were stationary, but the inner walls were rotational, while no-slip boundary condition was applied on them. There were interfaces between the stationary and the rotational regions. The rotating velocity of the impeller was considered to be 2500 rpm.

#### Near-wall treatment

The most popular way for considering wall effects is wall functions. The wall function method uses empirical formulas that impose suitable conditions near the wall without resolving the boundary layer. The major advantages of the wall function approach is that the high gradient shear layers near walls can be modeled with relatively coarse meshes. All turbulence models in CFX are suitable for a wall function method. The Low-Reynolds-Number method resolves the details of the boundary layer profile by using very small mesh length scales in the direction normal to the wall (very thin inflation layers). Turbulence models based on the $k-\omega$ equation, such as the SST model, are suitable for a Low-Reynolds-Number method. Low-Reynolds-Number approach requires a very fine mesh in the near-wall zone. Computer-storage and run-time requirements are higher than those of the wall-function approach. To reduce the resolution requirements the new wall boundary treatment is developed by CFX which switches automatically from a low-Reynolds number formulation to a wall function treatment based on grid density without a loss in accuracy^36^. The new wall boundary is introduced as Automatic wall-function which is chosen as wall function treatment in the present study.

#### Convergence criterion

**Figure 7:**
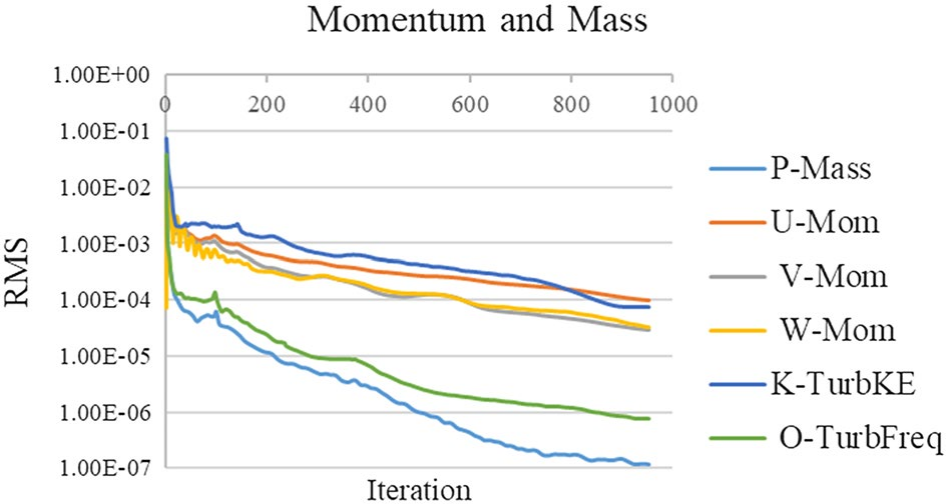
Pressure at the outlet in different iterations.

**Figure 8:**
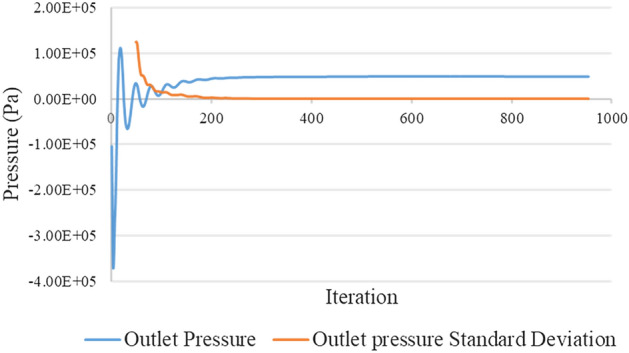
Standard deviation of the pressure changes at the outlet in different iterations.

The solution convergence was evaluated by setting the residual errors to 10^–4^ and monitoring the pressure at the outlet^71^. Figure 7 depicts RMS in different iterations. Figure 8 demonstrates the standard deviation of the pressure changes at the outlet for every 50 iterations. According to Fig. 6, when standard deviation is under 100 Pa (the value chosen in the present study), the pressures have 100 Pa variation from the mean value of the pressure at the outlet which helps in choosing proper number of iterations.

#### Solution method

To assess the LVAD performance, steady-state models utilizing the implicit finite element method were developed for different geometries. LVAD total head at a specific volume flow was calculated. HI was estimated by using calculated shear stresses and RBC exposure time. Wall shear stress contours were reported to analyze the high shear stress regions. RBC tracks were depicted using streamlines from the inlet to the outlet and averaging the time on streamlines represent the exposure time.

### Validation

**Figure 9:**
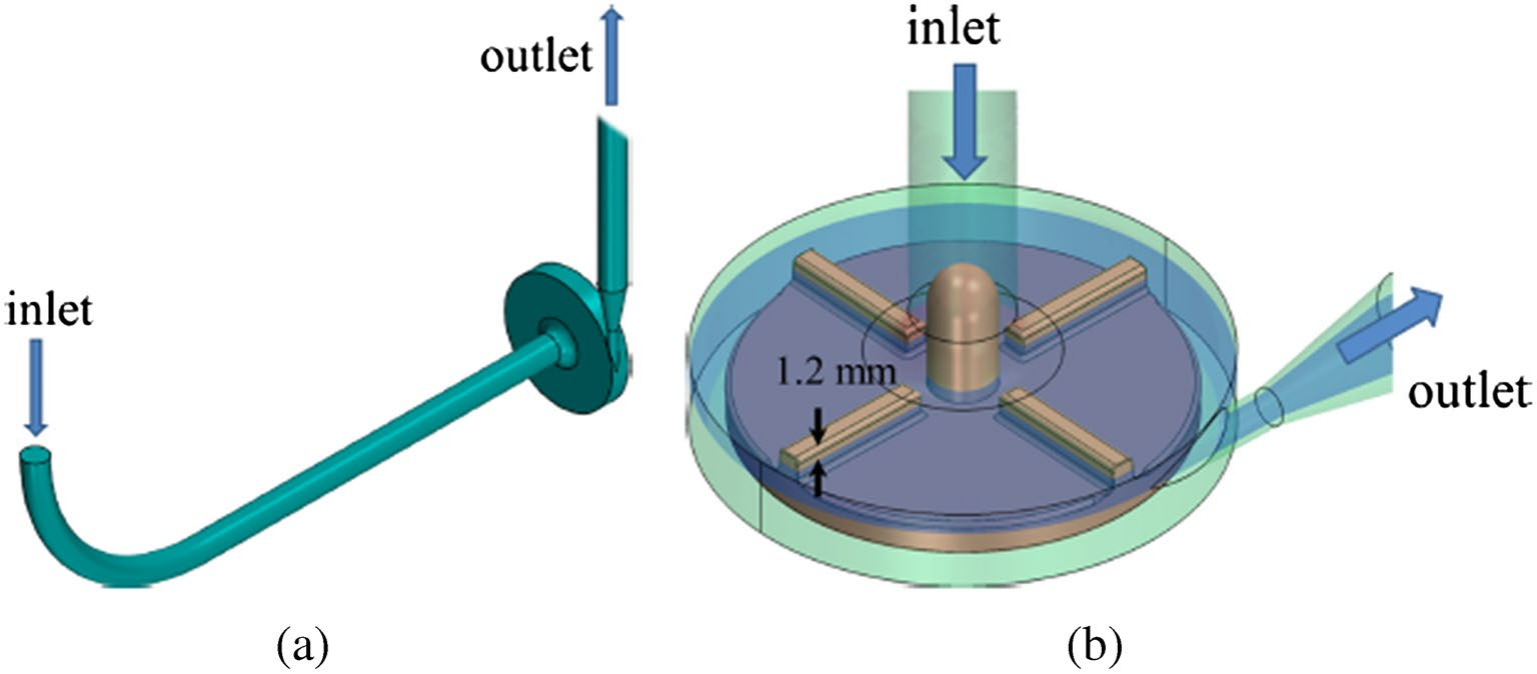
(**a**) Geometry of the FDA’s benchmark blood pump^53^. (**b**) Impeller zone (including the plane on which velocity field is investigated).

Due to the lack of standardized methods for validating CFD simulations and blood damage predictions, FDA initiates a benchmark blood pump to provide a comparison of numerical results with experimental data^28^. The FDA pump was tested in multiple laboratories to provide experimental data. Considering the FDA benchmark pump as a reference of accuracy of the CFD application, it was modeled using the same process of modeling described in “Numerical solution” section to validate present study CFD method. Figure 9 illustrates the FDA benchmark pump which is a centrifugal blood pump with four straight blades of height 3 mm. The operating conditions was an angular velocity of 3500 r/min with the flow rate of 6 l/min and velocity magnitude on a plane which is 1.2 mm below the top surface of blades was calculated.

**Figure 10:**
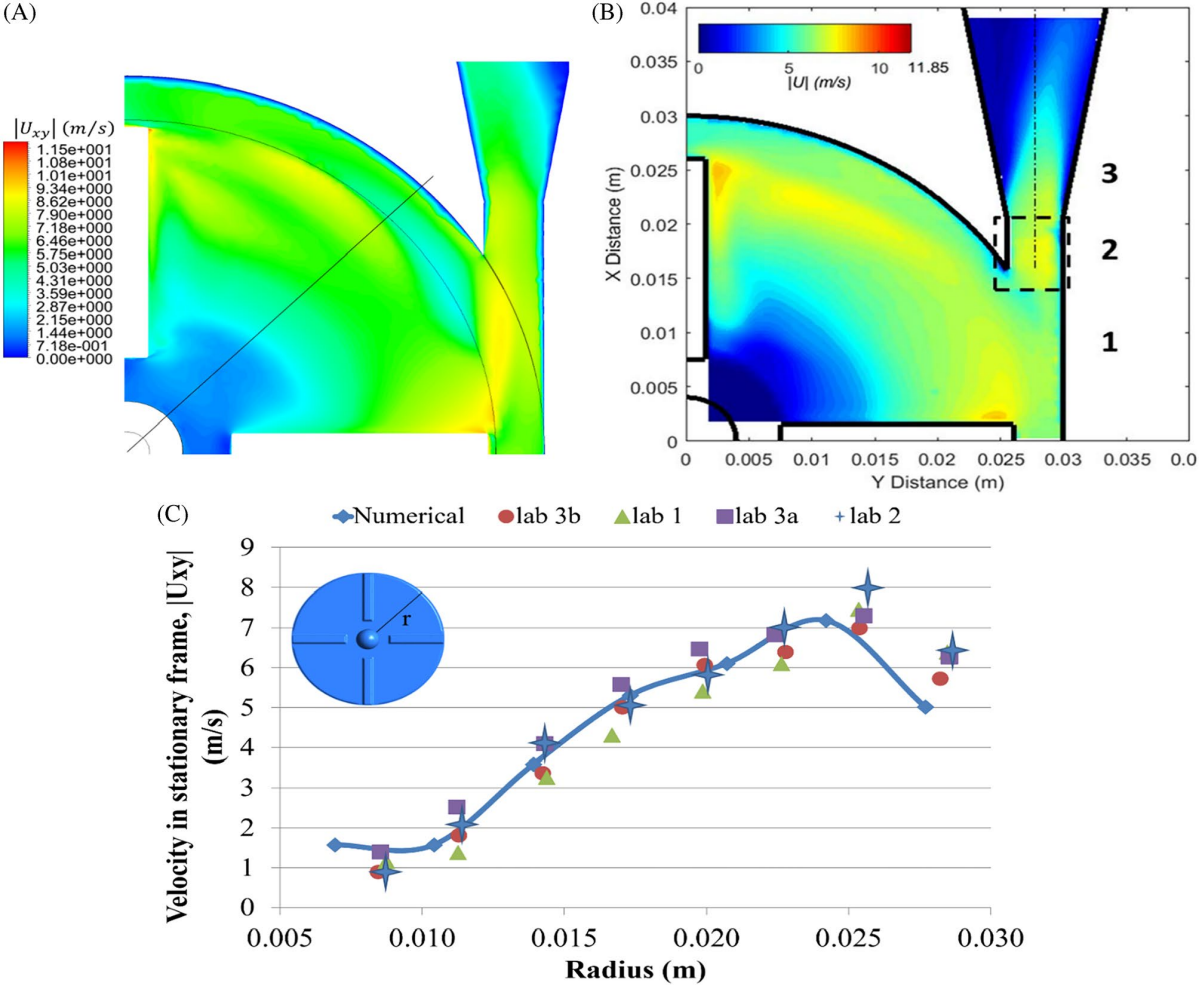
Speed comparison between simulated FDA blood pump and laboratory data; (**A**) velocity contour obtained from simulation; (**B**) velocity contour obtained from PIV^53^; (**C**) comparison of the velocity profile obtained from the simulation along the radius with the experimental data.

Figure 10 represents the quantitative comparison between numerical results of simulating FDA benchmark pump here and the experimental data provided. Maximum velocity magnitude was 8.7 ± 0.5 m/s reported from PIV by FDA, and 7.18 m/s obtained from numerical result, by which the variation between experimental measurement and numerical result was then calculated to be 17%. The lowest average variation of velocity magnitude between different laboratories measurements and the numerical results was calculated to be 8% (lab 3b) and the highest was 16% (lab 1) which indicates there is a reasonable agreement between experimental data and numerical results.

## Result

In this section, the performance of impellers designed by the point-by-point method will be investigated, and their results will be reported. The difference between these impellers is in the relationship between the angle of the blade and the radial position, the blade’s inlet angle, and the blade’s outlet angle.

Due to the high number of impellers in this section, only the blade’s total head and the blade’s hemolysis index graphs are reported. A 4-digit symbol was used to mark each blade, in which the first two digits indicate the blade’s inlet angle, and the second two digits indicate the blade’s outlet angle. For example, 4555 will indicate the inlet angle of 45° and the outlet angle of 55° for the blade. In addition, the letters L and S at the beginning of this numerical sign will indicate the use of a logarithmic and second order relationship between the angle of the blade and the radial position, respectively.

### Head

This part compares the calculated total head values for the logarithmic and second order impeller.

**Figure 11:**
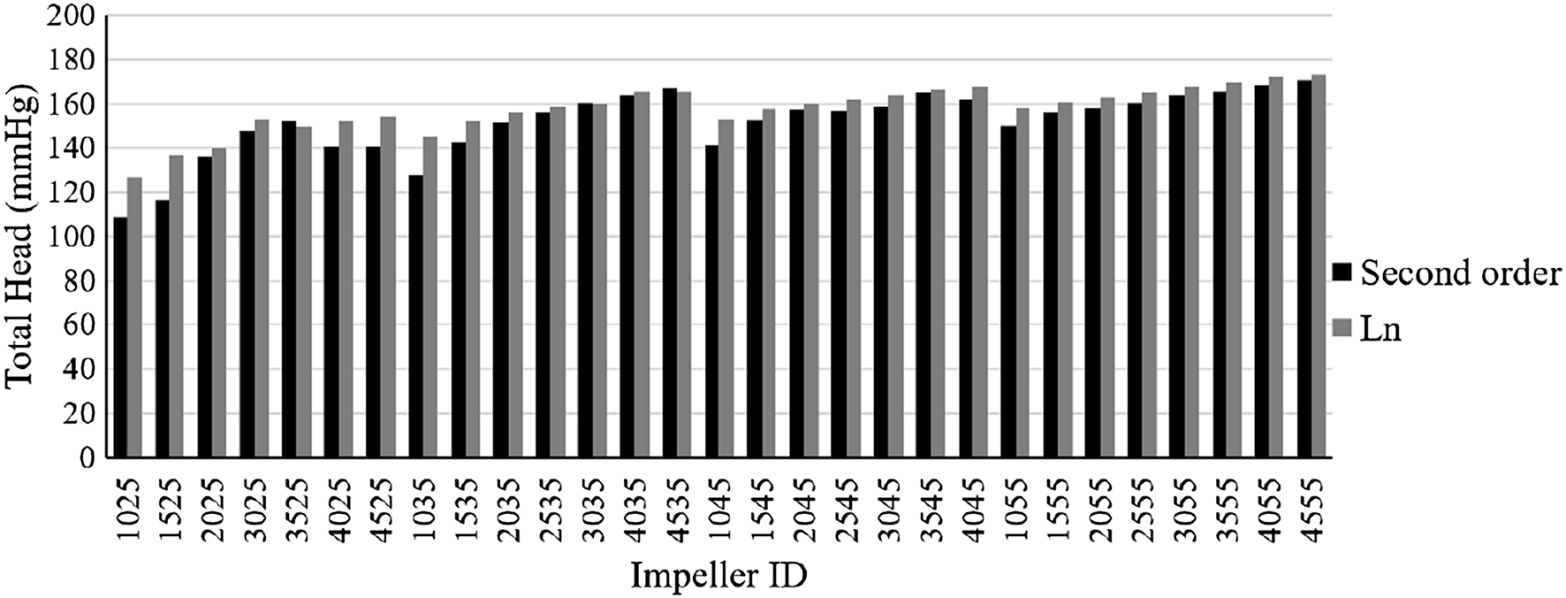
Total head values for blade types.

Figure 11 shows the Total head. As can be seen, in most cases, with the constant outlet angle, the calculated total head for the logarithmic impellers is greater than that of the second order impellers, and only in the 3525 and 4535 impellers, the produced head by the second order impeller is more than logarithmic impeller was obtained.

The highest difference between the production head was observed in the 1525 impeller, and the least was observed in the 3035 impeller. As it is known, the highest amount of production head among all impellers is reported in the L4555 impeller equal to 173 mmHg, and the lowest amount is reported for the S1025 impeller equal to 108 mmHg.

**Figure 12:**
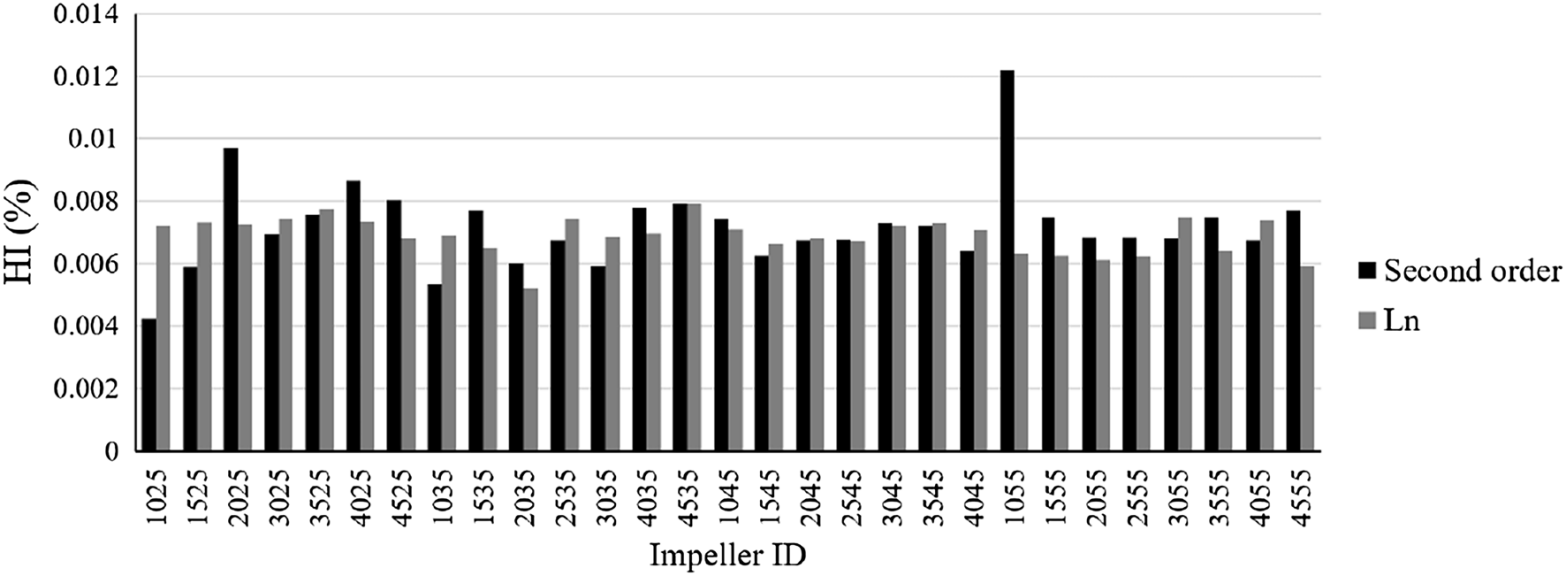
Hemolysis index for blade types.

Figure 12 shows the hemolysis index. The highest amount of calculated hemolysis is related to impeller S1055, and the lowest is related to S1025.

According to the results reported up to this section, the L4555 impeller has the highest head production among the other impellers, while this impeller is among the impellers with the lowest hemolysis index.

### Simulation results of L4555 impeller

**Figure 13:**
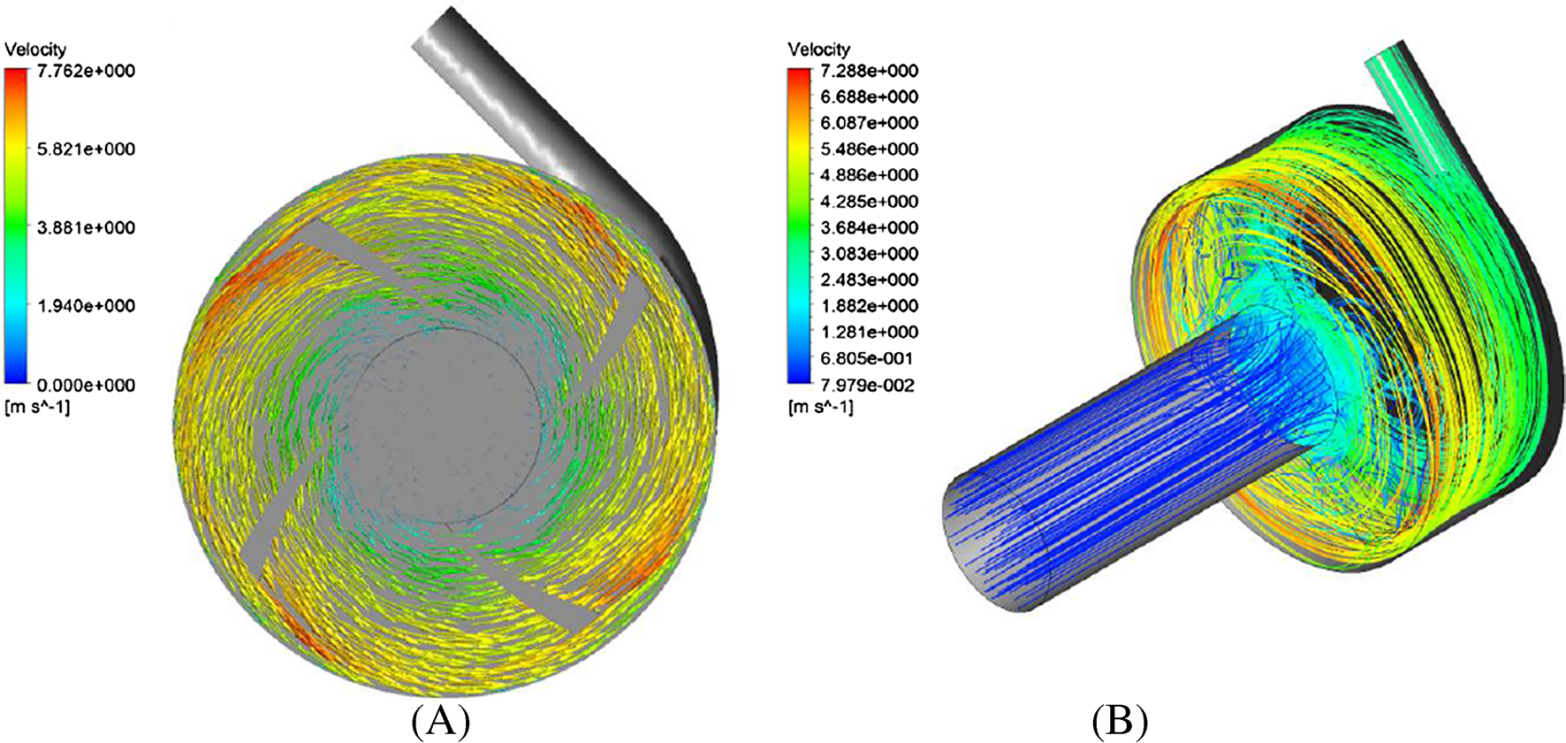
(**A**) Velocity vectors in the plane with a distance of 0.4 mm from the upper surface of impeller, (**B**) streamlines for L4555 impeller.

Figure 13 shows the velocity vectors (on the plane located at a distance of 2.5 mm from the upper surface of the impeller) and the streamlines. As shown in Fig. 13, the orientation of the velocity vectors is very uniform and no significant changes are observed in the pattern of the velocity vectors.

**Figure 14:**
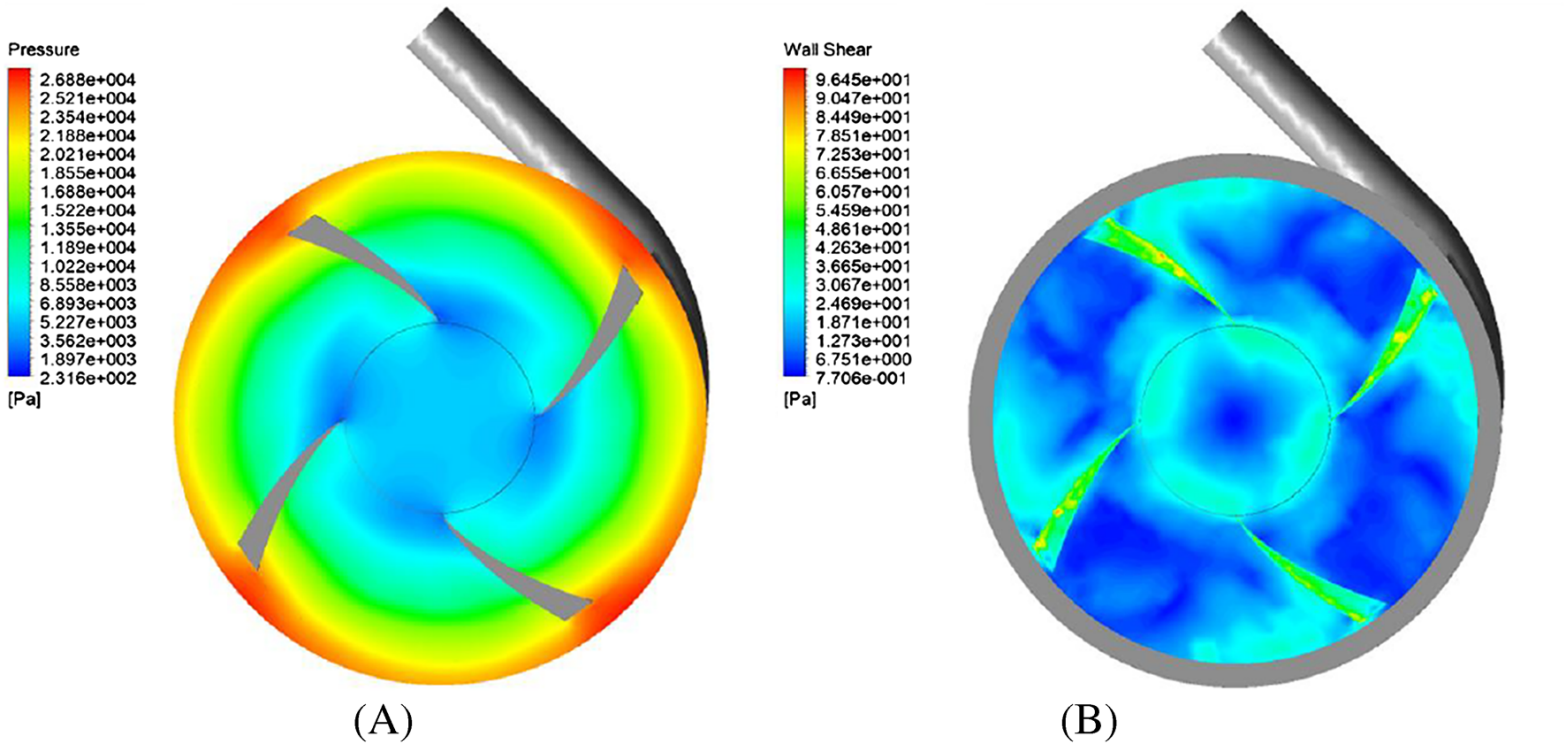
(**A**) Pressure contour in the plane with a distance of 0.4 mm from the upper surface of impeller. (**B**) Wall shear contour for L4555 impeller.

Figure 14 shows the pressure contour (on a plane located at a distance of 2.5 mm from the upper surface of the impeller) and wall shear stress contour. According to Fig. 14A, the pressure increased in the radial direction as expected. Figure 14B shows the wall shear stress contour on the impeller. As can be seen, the wall shear stress on the impeller is always less than 100 Pa.

In this section, the L4555 impeller was modified and studied for use in the FDA standard pump. Velocity vectors, streamlines, and wall shear stress contours were compared from the simulation of both impellers in the FDA standard pump body. For the L4555 impeller, the performance curve obtained from the simulation and experimental results were reported. Finally, the performance curve obtained from the experimental test of the impeller designed in this study was compared with the FDA standard pump impeller.

### The results of the numerical comparison of the designed impeller in the present study with the impeller used in the FDA pump

This section reports parameters, including velocity vectors, streamlines, and wall shear stress contours on the impeller. The results are compared between the L4555 impeller and the impeller used in the FDA pump. Finally, the performance curve obtained from the simulation of the designed impeller in this study is compared with the experimental test of the same impeller.

### Streamlines and velocity vectors

**Figure 15:**
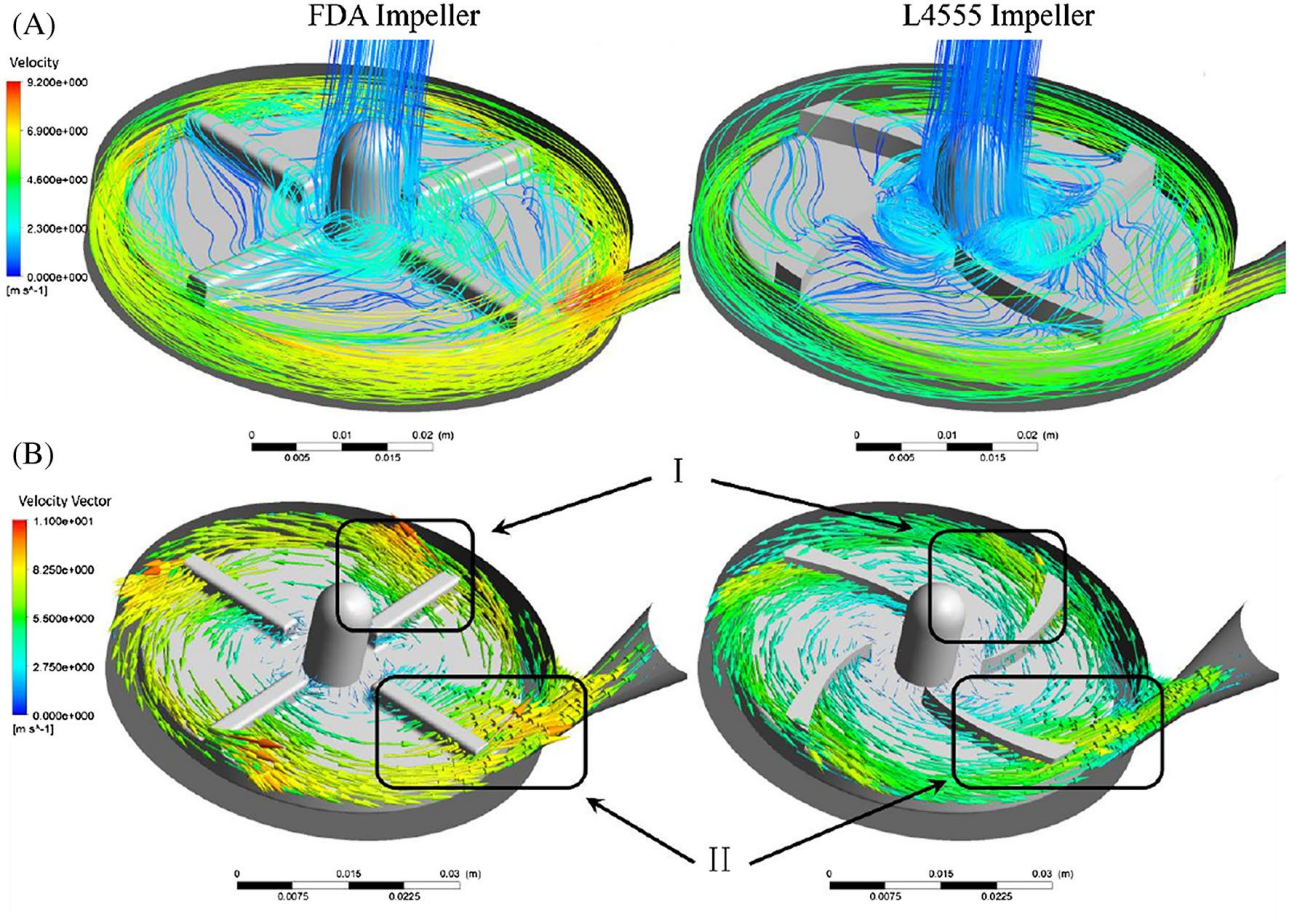
(**A**) Streamlines, (**B**) velocity vectors for L4555 impeller and FDA impeller.

Figure 15 shows the streamlines and velocity vectors. As can be seen, the streamlines for the FDA impeller, compared to the designed impeller in the present study, have higher congestion in the pump volute chamber, indicating that the fluid will remain in the pump for a longer time.

**Figure 16:**
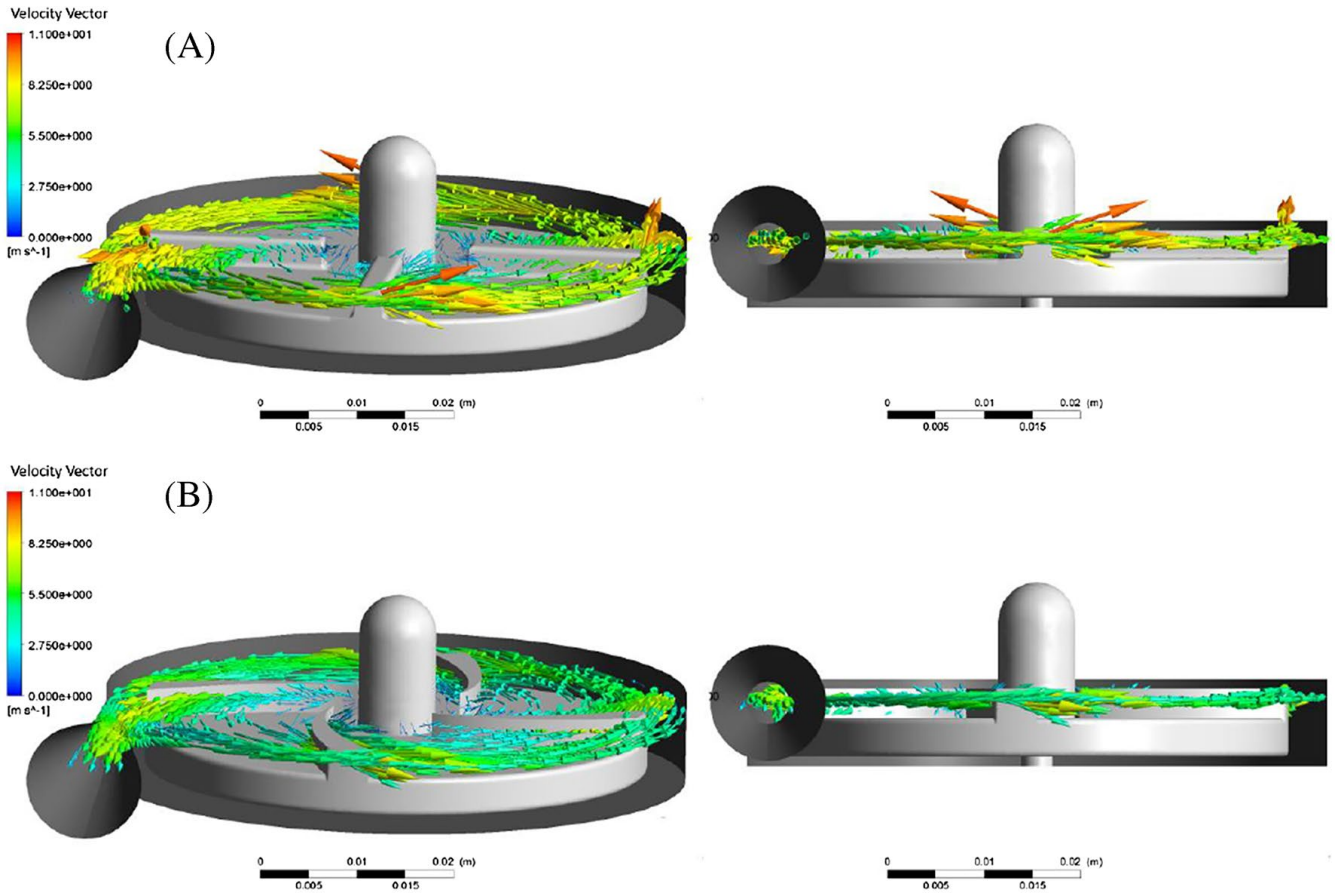
The dispersion of velocity vectors (**A**) FDA impeller, (**B**) L4555 impeller.

Figure 15 shows the velocity vectors in a plane that is 1.2 mm from the upper surface of the blade. As seen in the (I) and (II) areas, the radial components of the velocity vector in the designed impeller in this study have larger values than the FDA impeller. In general, it can be seen that the velocity vectors in the designed impeller in this research have a more regular pattern than the FDA impeller. The velocity vectors at the pump outlet are more uniform for the designed impeller in this study. As shown in Fig. 16, the velocity vectors in the designed impeller in this study have fewer components perpendicular to the velocity vector drawing plane.

### Wall shear stress contour

**Figure 17:**
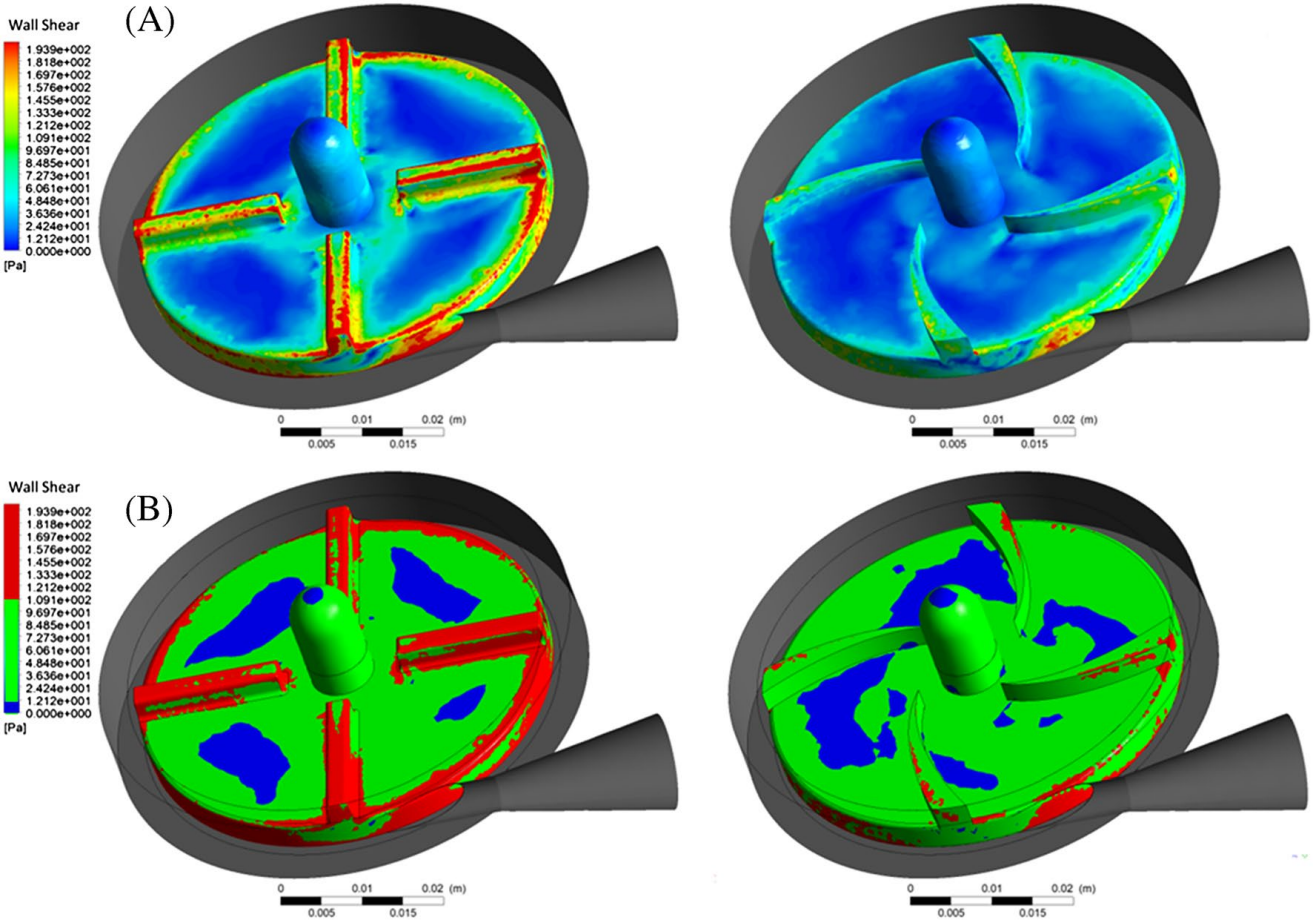
(**A**) Shear stress contour, (**B**) shear stress in the range less than 10 (Pa) (blue color), between 10 to 100 (Pa) (green color) and more than 100 (Pa) (red color).

Figure 17A shows the wall shear stress contour. Figure 17 shows that the wall shear stress on the FDA impeller has higher values than the designed impeller in the present study. Generally, calculated wall shear stress on the edges of the impeller hub and the impeller blades show high values.

Figure 17B shows the wall shear stress in three ranges: Less than 10 Pa in blue color, between (10 and 100 Pa) in green color, and above 100 Pa in red color.

Wall shear stresses less than 10 Pa are within the range of physiological stresses. The wall shear stress between (10 Pa) and (100 Pa) leads to the reduction of the von Willebrand factor and, ultimately platelet activation. The continued presence of active platelets stimulates the formation of thrombosis.

Stresses higher than 100 Pa significantly increase the possibility of hemolysis. As shown in Fig. 17B, in the FDA impeller, compared to the designed impeller in this study, more areas experience wall shear stress higher than 100 Pa.

The area where the shear stress is less than 10 Pa occupies a smaller area in the FDA impeller. In general, the designed impeller in the present study shows better performance in terms of damage to the blood.

### Comparison of the performance curve obtained from the results of simulation and experimental test for the designed impeller in the present study

**Figure 18:**
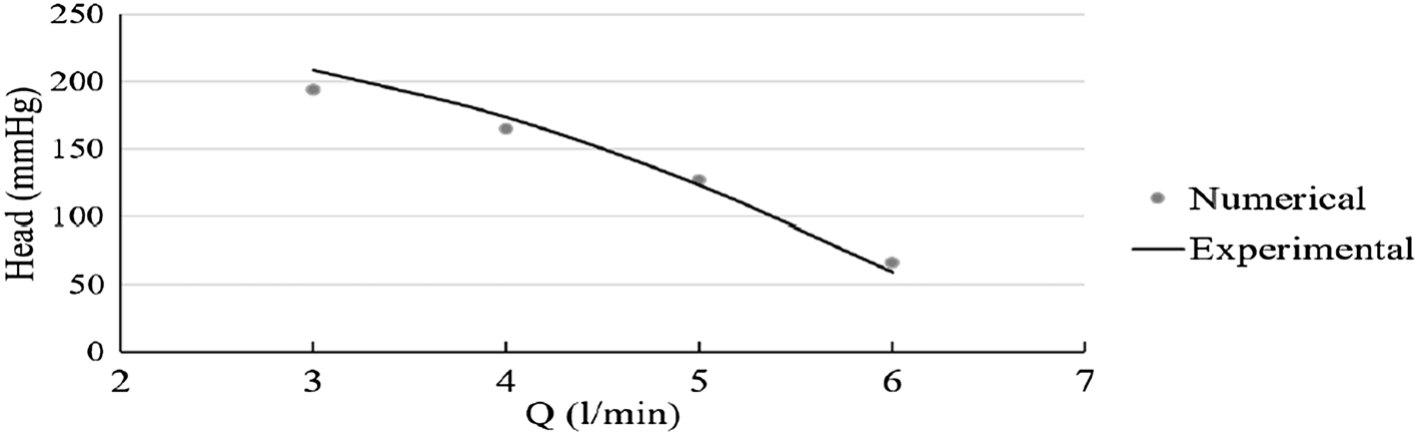
Performance curve obtained from simulation and experimental results at a speed of 2500 rpm.

Figure 18 shows Values related to the performance curve obtained from simulation and experimental results at a speed of 2500 rpm. As is apparent in the figure, the simulation results are in good agreement with the experimental test results.

### Comparison of the performance curve of the designed impeller in the present study with the FDA impeller

**Figure 19:**
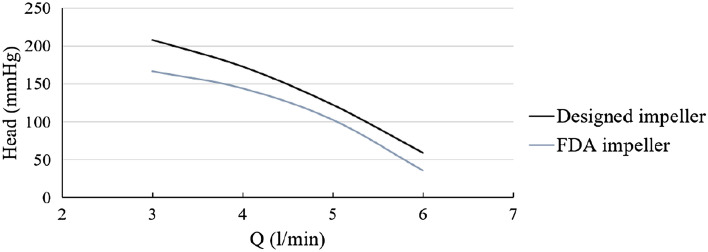
Comparative diagram of the performance curve of the designed impeller in the present study and the FDA impeller.

Figure 19 shows the comparative diagram of the performance curve of the designed impeller in the present study and the FDA impeller, which results from an experimental test at a rotational speed of 2500 rpm. As evident in the diagram, the designed impeller in this study has a better performance, and at the design point (the flow rate (5 Lit/min)) increases the total head by almost 20%.

The simulation of impeller performance with different inlet and outlet angles shows that increasing the inlet and outlet angle will often increase the total head.

The results show that the designed impellers using the logarithmic function create a higher total head than those with the second order function. Also, the hemolysis index calculated for all the impellers is within an acceptable range for There are clinical applications. Among all the impellers studied in this study, the L4555 impeller has the best performance.

Then, the experimental performance of the L4555 impeller was compared with the FDA impeller, both located inside the FDA volute chamber. The results indicated that the performance of this impeller compared to the FDA impeller increases by at least 20% in the total head at different flow rates.

**Figure 20:**
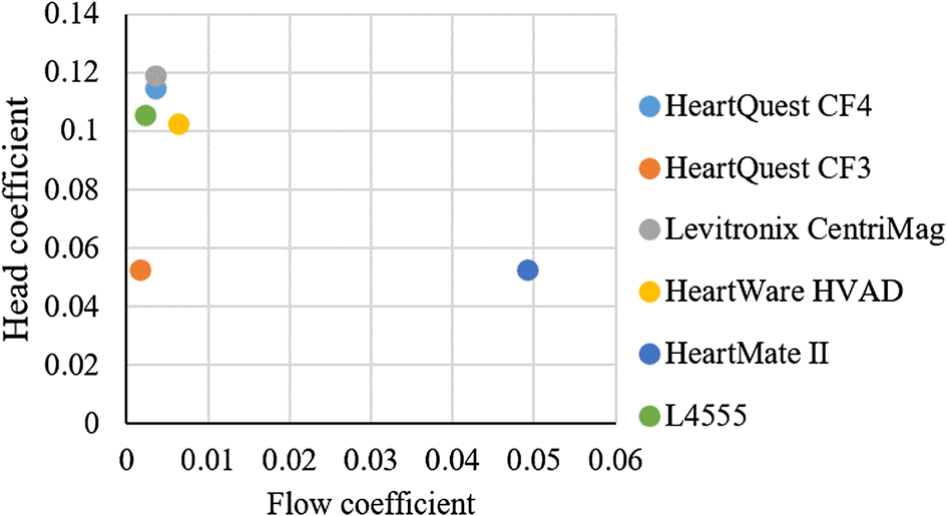
Values of the flow coefficient and height coefficient for the pump designed in this study and some pumps used as ventricular assist pumps.

Figure 20 shows the values of the flow coefficient and height coefficient for the pump designed in this study and some pumps used as ventricular assist pumps. As can be seen in the figure, the designed pump is within the operating range of the HeartMate2, HVAD, and Levitronix CentriMag.
